# Residual radiopharmaceutical activity in venous access devices during PET and SPECT imaging: a real-world clinical observational cohort study

**DOI:** 10.1186/s41824-026-00317-4

**Published:** 2026-09-01

**Authors:** Rasmus Solem, Nourhan Al-Amiri, Ida Skarping

**Affiliations:** 1 Radiation Physics, Department of Hematology, Oncology and Radiation Physics, Skåne University Hospital, Lund, Sweden; 2 Department of Clinical Physiology and Nuclear Medicine, Skåne University Hospital, Lund, Sweden; 3 Department of Oncology, Clinical Sciences, Lund University, Lund, Sweden

**Keywords:** PET, SPECT, Radiopharmaceutical, Intravenous access site, Venous access points, Peripheral intravenous catheter, Central intravenous catheter, Residual activity fraction

## Abstract

**Background:**

In routine practice, radiopharmaceuticals are administered via a newly placed peripheral intravenous catheter (PIVC). However, many patients referred for nuclear medicine imaging already have a semipermanent venous access device in place, such as a central venous catheter, peripherally inserted central catheter, or subcutaneous implantable venous port. Evidence regarding residual radiopharmaceutical activity within preexisting devices—and its potential consequences for SPECT/PET image quality, diagnostic interpretation, and radiation safety—remains limited. This study aimed to quantify device-related residual radiopharmaceutical activity across commonly used access types and to assess any associated clinical impact during image review.

**Results:**

In this observational cohort study at Skåne University Hospital, Sweden, 167 patient administrations during SPECT or PET imaging between June 2023 and January 2026 were included (40% aged < 18 years). Residual activity was evaluated using ROI/VOI based quantification at the venous access site on reconstructed or planar images. Measurements were performed in an activity meter using PIVCs that had been removed from patients prior to imaging. In an experimental setting, representative venous access devices were flushed under controlled conditions to determine residual activity fraction. Residual activity was expressed both as absolute activity (MBq) and as the percentage of the administered activity. Across 214 measurements including 13 radiopharmaceuticals, residual activity was generally low: only 3.3% of the measurements (7/214) exceeded 5% of the administered activity. The highest absolute residual activity was 31 MBq ([^99m^Tc]pertechnetate, PIVC), and the highest proportional residual activity was 17.3% ([^99m^Tc]Tc-MAA, Midline). On clinical image review, no instance of device related residual activity was associated with impaired interpretation or reduced diagnostic confidence.

**Conclusions:**

In this real-world cohort, residual radiopharmaceutical activity within preexisting venous access devices was generally low and showed no discernible impact on visual clinical SPECT or PET interpretation. Prospective studies including a broader range of tracers, access devices, and quantitative endpoints are needed to more fully characterize the clinical implications of residual activity.

**Supplementary Information:**

The online version contains supplementary material available at https://doi.org/10.1186/s41824-026-00317-4.

## Introduction

Radiopharmaceuticals are drugs that contain radionuclides, emitting radiation. Their distribution within the body depends on various factors, including physicochemical properties, radiolabeling stability, and patient pathophysiology. There are various routes of administration for radiopharmaceuticals, including oral, inhaled, intratumoral-, and subcutaneous injection; however, intravenous administration is most commonly used in nuclear medicine diagnostics. Thus, most adult and pediatric patients undergoing a gamma/single-photon emission computed tomography (SPECT) or positron emission tomography (PET) imaging procedure need a vascular access route for intravenous administration of the radiopharmaceutical. Usually, patients are provided with a peripheral intravenous catheter (PIVC) upon arrival at the nuclear medicine department. However, some of the patients, both in- and out-hospital patients, have a semi-permanent intravenous access site, there among peripherally inserted central catheters (PICCs), implanted venous ports, central lines, and dialysis catheters. The available literature of the recommendation and the safety of using these venous access devices for radiopharmaceutical administration in nuclear medicine is sparse and general guidelines do not address the questions of different access routes (Weatherman et al. 2025).

The amount of a radiopharmaceutical that can retain in venous access devices vary based on several factors, including the type of device, the technique used for administration, and the specific radiopharmaceutical. A small pre-clinical exclusively experimental study showed < 1% retained activity for tested vascular port models for [^18^F]FDG and Na[^18^F]F (Gossman et al. 2017). On the contrary, due to the adhesive nature of [^99m^Tc]Tc-MAA used in lung scintigraphy, in clinical practice it is deferred from injecting in an indwelling line or port that contains a filter, e.g. a PICC-line (Parker et al. 2012). For the authorized and marketed radiopharmaceuticals and preparation kits, the summary of product characteristics sometimes provides instructions on how the medicinal product should be prepared and administered. Apart from this, there is very sparse literature on radiopharmaceuticals and venous access devices.

High image quality and diagnostic accuracy are fundamentally dependent on the delivery of the intended radiotracer dose. The administered activity is typically determined by balancing radiation exposure against the requirements for adequate image quality, keeping radiation exposure as low as reasonably achievable known as the ALARA principle (European Association of Nuclear Medicine 2016, Boellaard et al. 2015).

Different mechanistic properties of the tracer may influence residual activity fractions: hydrophilic small molecules (e.g., [^18^F]FDG, [^99m^Tc]Tc-HDP) will theoretically clear quickly, while larger or charged molecules (e.g., peptides, antibodies, and nanoparticles) may linger if not flushed efficiently (Abou et al. 2015, Peterson and Manning 2009, Vallabhajosula 2009). Also, injection techniques in terms of speed and NaCl 0.9% flush volume might modify results.

Despite the recognized impact of injection quality on imaging, the effect of intravenous access devices on residual radiopharmaceutical activity and systemic delivery remains underexplored. The aim of this study was to investigate residual radiopharmaceutical activity in vascular access devices, including cannulas and implanted venous ports, during SPECT and PET imaging in both routine clinical practice, and in an experimental laboratory setting designed to isolate device-related residual activity under controlled conditions. In addition, with a study focus on optimizing clinical practice and clinical workflow, importantly, a study aim was to evaluate the implication of clinical interpretation of the scan if residual activity was present at the intravenous access site. Note that further evaluation of pharmacodynamics was out of scope of this study.

## Methods

### Study population

**Fig. 1:**
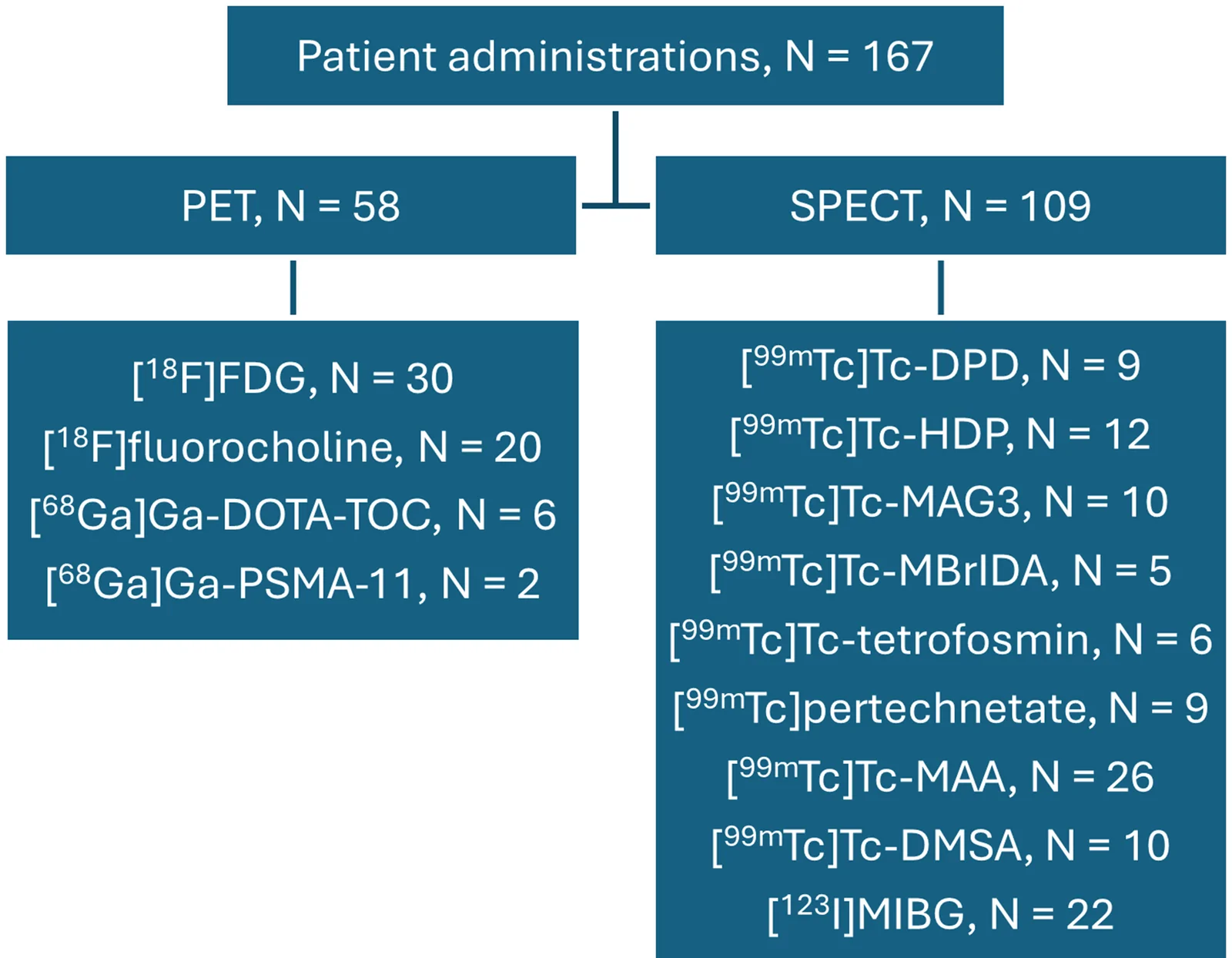
Flowchart over patient administrations for the 13 different radiopharmaceuticals included in this study

The study was conducted at the Department of Clinical Physiology and Nuclear Medicine, Skåne University Hospital in Lund and Malmö, Sweden, between June 2023 and January 2026, and included a total of 167 patient administrations (Fig. 1), of which 67 (40%) were performed on patients under 18 years of age. Patients undergoing multiple examinations could contribute more than one scan, as each scan was treated as an independent observation. To mirror the clinical settings, inclusion criteria were broad, and patients fulfilling these two criteria were eligible: (1) Patients of all ages referred to a gamma/SPECT/PET procedure and (2) Patients administered any radiopharmaceutical intravenously. For inclusion, the venous access device had to be included in the images retrieved or an extra image over the system had to be acquired, i.e. the requirements of calculating the residual activity fraction. Predominantly patients with an indwelling semi-permanent venous access device were invited to participate. Patients undergoing imaging with standard PIVCs were additionally included to enable comparison. All injections were administered via clinically indicated venous access routes per standard procedure.

A total of 68 patient administrations were retrospectively identified through the hospital Picture Archiving and Communication System (PACS) and included in the study under Ethics Board waiver.

### Radiation safety considerations

For all patients included in the study who underwent PET examinations, the injection site was visible on the clinical scans. Patients referred to planar gamma camera or SPECT examinations, and where the injection site was not included in the clinical field of view, a static planar image was acquired. Hence, no patients received any extra radiation exposure for their inclusion in this study.

### Venous access device and NaCl 0.9% flush

As per clinical routine and in accordance with technologist guideline, when venous access device was provided at the imaging department, a direct venepuncture (straight stick) was performed, predominantly of veins in areas of flexion (antecubital fossa, hand, and wrist) for intravenous access placement. Only PIVCs and 3-way stopcocks were available for insertion at the department, all other venous access systems were already in use before patient arrival. Various vascular access devices used in the clinic were evaluated separately, including: PIVCs (of different calibers and lengths, the latter i.e. Deep Access and Midline), central venous catheter (CVC), PICCs, Nexiva™ Closed IV Catheter System, and implanted venous ports. An illustrative overview of the included venous access devices and representative vascular access routes is provided in Supplementary Material 1.

As per general local clinical standard (Region Skåne, Vårdhandboken 2026), i.e. no tracer-specific practical recommendations, the venous access device was flushed with NaCl 0.9% before and after radiopharmaceutical administration (Table 1). During [^18^F]FDG and [^68^Ga]Ga-DOTA-TOC examinations where a diagnostic computed tomography (CT) with intravenous contrast was acquired, the venous access devices were flushed with up to 245 ml NaCl 0.9% and contrast agent combined. For some patients, [^18^F]FDG was administered using the automated injector system Medrad Intego (Bayer Healthcare, Germany).

**Table 1:** Standard NaCl 0.9% flush volumes in routine clinical practice after radiopharmaceutical administration

Radiopharmaceutical	Standard NaCl 0.9% flush volume (ml)
[^99m^Tc]Tc-DMSA	5–10
[^99m^Tc]Tc-DPD	10
[^99m^Tc]Tc-HDP	10
[^99m^Tc]Tc-MAA	20
[^99m^Tc]Tc-MAG3	15
[^99m^Tc]Tc-MBrIDA	10
[^99m^Tc]pertechnetate	10
[^99m^Tc]Tc-tetrofosmin	10
[^123^I]MIBG	20–40
[^18^F]FDG with automatic injector	35
All PET-tracers manual injections	10

### Evaluating residual radiopharmaceutical activity

#### Evaluation based on imaging

Regions-of-Interest (ROIs) or Volumes-of-Interest (VOIs) were manually drawn over the venous access devices and lines using the standard clinical software HERMIA GoldLx (Hermes Medical Solutions, Stockholm, Sweden). Counts measured within the ROIs/VOIs of SPECT and static images were converted to activity using system specific calibration factors, whereas the PET systems were directly cross calibrated to an activity meter. The decay-corrected residual activity was determined considering background activity.

#### Quantification based on direct radioactivity measurements

The residual activity for each removable PIVC was measured in a VIK-202 5051 (Comecer Netherlands B.V., Joure, Netherlands) activity meter and corrected for background activity and radioactive decay. Isotope-specific calibration factors, calculated to account for a variety of vials and syringe geometries, were applied for the measurements.

#### Residual activity fraction

The residual activity fraction was calculated as $\:\frac{Residual\:Activity\:\left[MBq\right]}{Administered\:Activity\:\left[MBq\right]}$ where the administered activity was corrected for activity retained in the syringe. Following radiopharmaceutical administration, the syringe was flushed with NaCl 0.9% per local clinical standard. After the syringe had been removed, the decay corrected residual activity in the emptied syringe was measured in the activity meter.

### Assessment of impact on clinical image interpretation

To assess the potential impact of residual radiopharmaceutical activity on clinical image interpretation, examinations were reviewed for visually detectable activity at the venous access site, including along the vascular access route, and at the external device location. Assessment was restricted to studies in which the venous access device was within the clinical field of view.

Interference with image interpretation was defined as device related activity that could not be clearly localized to the access device itself and therefore risked misinterpretation as pathological uptake. Diagnostic confidence was evaluated based on the ability to interpret the examination without reduced lesion conspicuity or perceived image quality.

Study-specific image review was performed by an experienced reader (IS). Additionally, routine clinical reports were reviewed for documentation of any device related interpretative difficulties.

### Experimental set up

Venous access devices used in the clinic were used to manually inject the radiopharmaceutical into a lead shielded glass vial. Activity levels resembling the clinical examinations were used and cannulas were flushed with NaCl 0.9% mirroring clinical routine. Decay corrected residual activity in cannulas and stopcocks were measured separately in an activity meter and the residual activity fraction was calculated as the fraction of the administered activity.

### Statistical analyses

Differences in residual activity in venous access devices were evaluated for each radiopharmaceutical and each type of venous access device. Given the many different combinations, the statistics are to be regarded as exploratory and no a priori difference in clinically relevant difference was established. Although PIVCs are generally considered the optimal venous access system and benefit from substantial clinical experience, they were not used as a reference standard for non‑inferiority comparisons in this study. Consequently, residual radiopharmaceutical activity was not assessed relative to PIVCs. Available study participants within a reasonable time and at the expenditure of reasonable resources were included, thus, no power calculations were performed. A threshold of 10% difference between prescribed and administered activity was applied, reflecting a commonly accepted pragmatic cutoff in routine nuclear medicine practice and considered potentially clinically relevant with respect to adequate administered activity, imaging quality, and patient safety (Weatherman et al. 2025). As residual activity was generally low, an additional cutoff of 5% was applied.

Statistical analyses and graphical presentations were performed using Microsoft Excel (Microsoft Corporation, Redmond, WA, USA) and Python (Python Software Foundation, Wilmington, DE, USA).

### Literature search

To identify relevant studies, a systematic literature search was conducted across databases, including PubMed, Embase, and INSPEC. The search strategy was developed in collaboration with a medical librarian and included a combination of MeSH terms and keywords related to the research topic based on PICO assessment of the research question(s) at hand (Supplementary Material 2). The search terms were tailored to each database to maximize the retrieval of relevant articles. The initial search yielded 240 articles. After removing duplicates (*n* = 13), 227 articles remained. Titles and abstracts were screened independently by a single reviewer (IS) to assess their relevance. Full-text articles were then retrieved and assessed for eligibility based on their relevance; resulting in a total of seven articles (two case reports (Becherer et al. 1998, Slavin et al. 2004), three conference abstracts (Jacob 2022, Sunderland et al. 2024, Warminski et al. 2019), two full text articles (Gossman et al. 2017, Drescher et al. 2021). In addition, one conference abstract was identified through review of reference lists of the identified articles (Miozza et al. 2024) and one full text article based on an identified conference abstract (Sunderland et al. 2023).

## Results

A total of 167 patient administrations from 144 unique patients were included, covering 13 different radiopharmaceuticals (Fig. 1). Repeated examinations from the same patient were treated as independent observations; 23 patients contributed more than one examination. In some cases, measurements were obtained from more than one component (injection device, stopcock, and injection membrane), resulting in 214 measurements. Of these, 130 administrations were evaluated by direct radioactivity measurements of removable PIVCs using an activity meter, whereas 84 were assessed retrospectively by image-based ROI/VOI analysis.

### Residual activity fraction

**Fig. 2:**
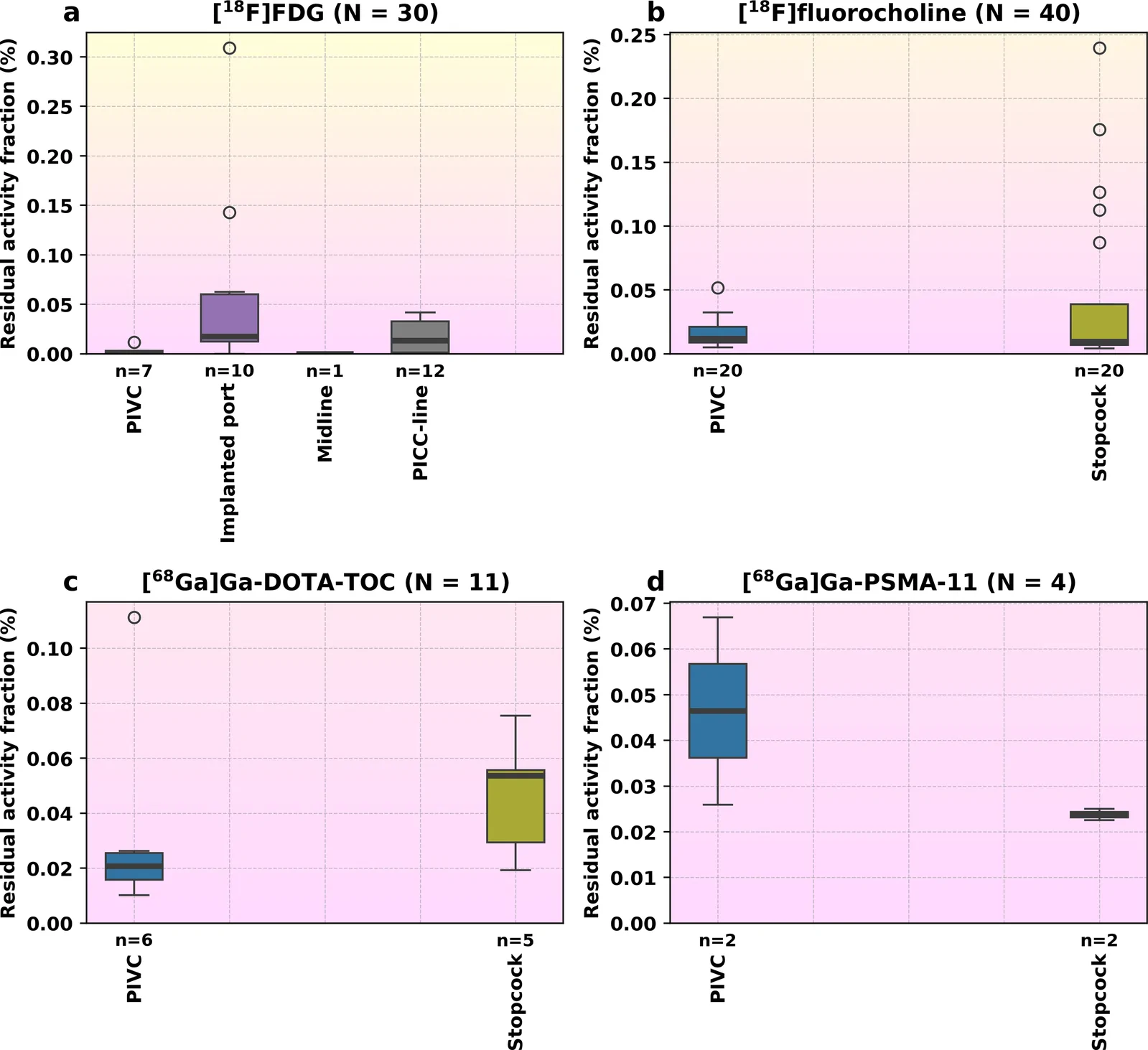
Residual activity fraction for the PET radiopharmaceuticals (**a**-**d**) for patients administrations. Note the different range of the y-axes of the subplots, highlighted by the color gradient background ranging from zero to the global maximum of the figure

**Fig. 3:**
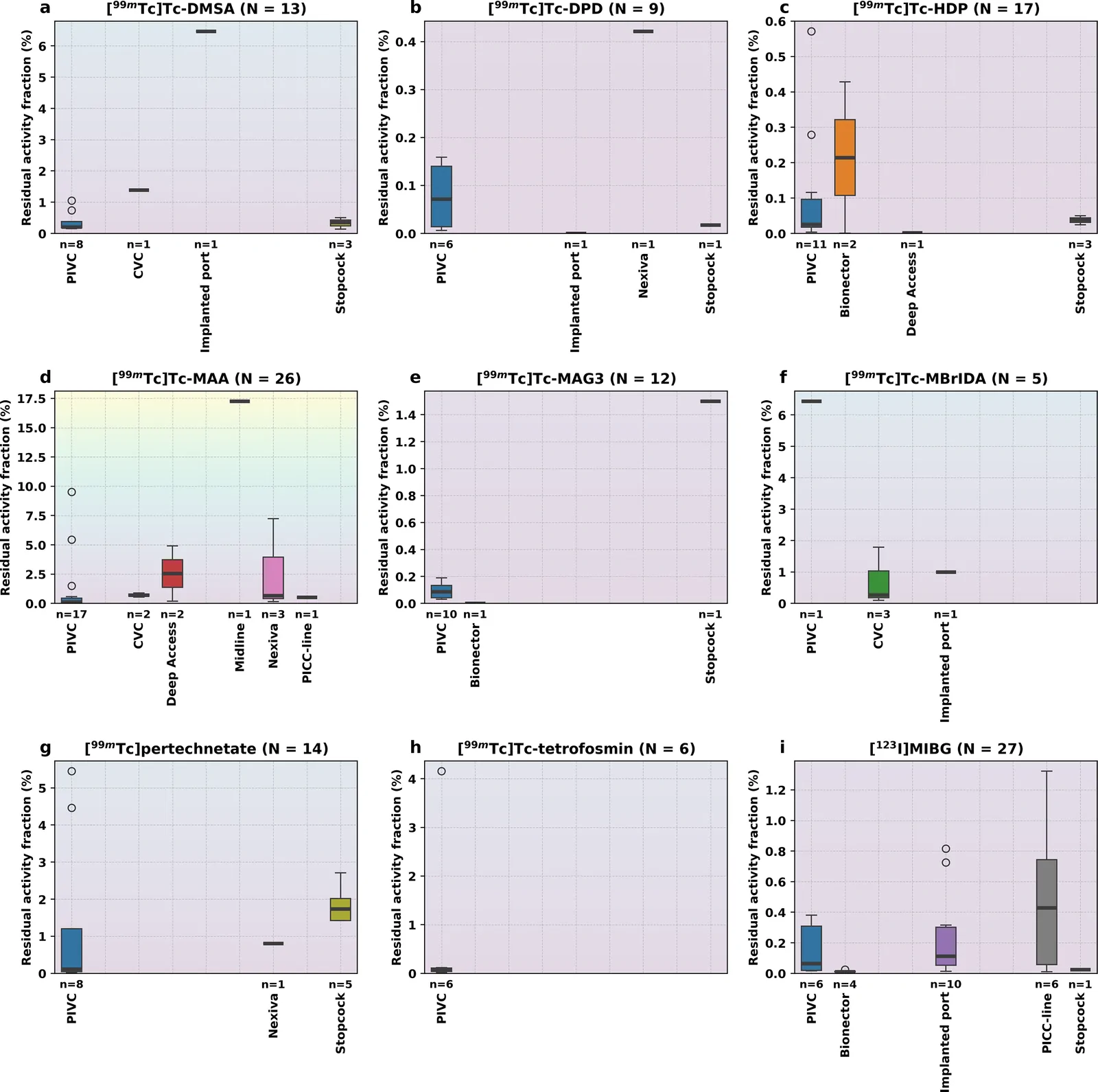
Residual activity fraction for the SPECT radiopharmaceuticals (**a**-**i**) for patients administrations. Note the different range of the y-axes of the sublots, highlighted by the color gradient background ranging from zero to the global maximum of the figure

The residual activity fractions were generally low for all SPECT and PET radiopharmaceuticals and are visually summarized in Figs. 2 and 3. The corresponding datasets for clinical measurements are outlined in Supplementary Material 3a.

For the four PET radiopharmaceuticals, the range of residual activity fractions across the patient measurements were ≤ 0.3% (*n* = 30), ≤ 0.2% (*n* = 40), ≤ 0.1% (*n* = 11), and ≤ 0.1% (*n* = 4) for [^18^F]FDG, [^18^F]fluorocholine, [^68^Ga]Ga-DOTA-TOC, and [^68^Ga]Ga-PSMA-11, respectively.

Among the SPECT radiopharmaceuticals, the residual activity fraction did not exceed 1% for [^99m^Tc]Tc-DPD and [^99m^Tc]Tc-HDP. For [^123^I]MIBG, [^99m^Tc]Tc-MAG3, [^99m^Tc]Tc-tetrofosmin, [^99m^Tc]pertechnetate, [^99m^Tc]Tc-MBrIDA, and [^99m^Tc]Tc-DMSA, the highest residual activity fraction remained below 7%. Residual activity fractions exceeding 10% were observed in only one instance of [^99m^Tc]Tc-MAA (17.3%, Midline).

**Fig. 4:**
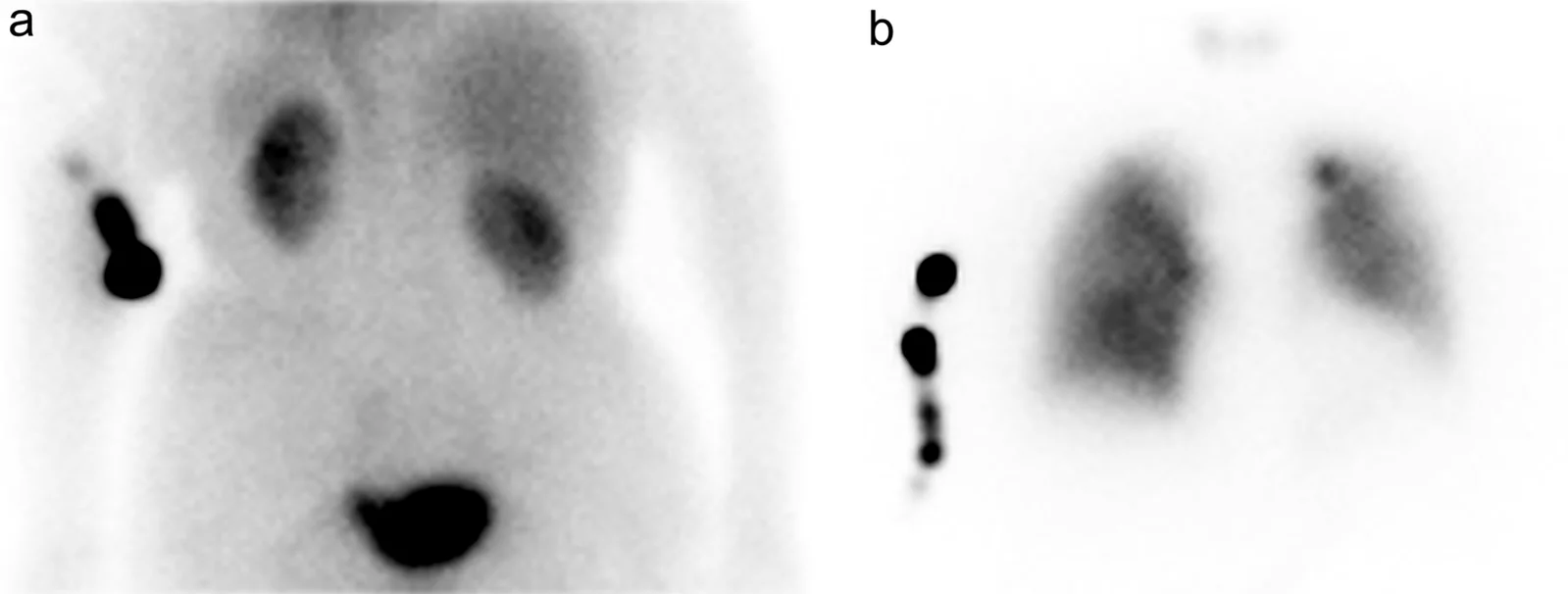
Patient images showing **a**) the highest measured residual activity of 31 MBq (4.5% of administered activity) of [^99m^Tc]pertechnetate in a peripheral intravenous catheter and **b**) the highest measured residual activity fraction of 17.3% (19 MBq) of [^99m^Tc]Tc-MAA in a midline

**Fig. 5:**
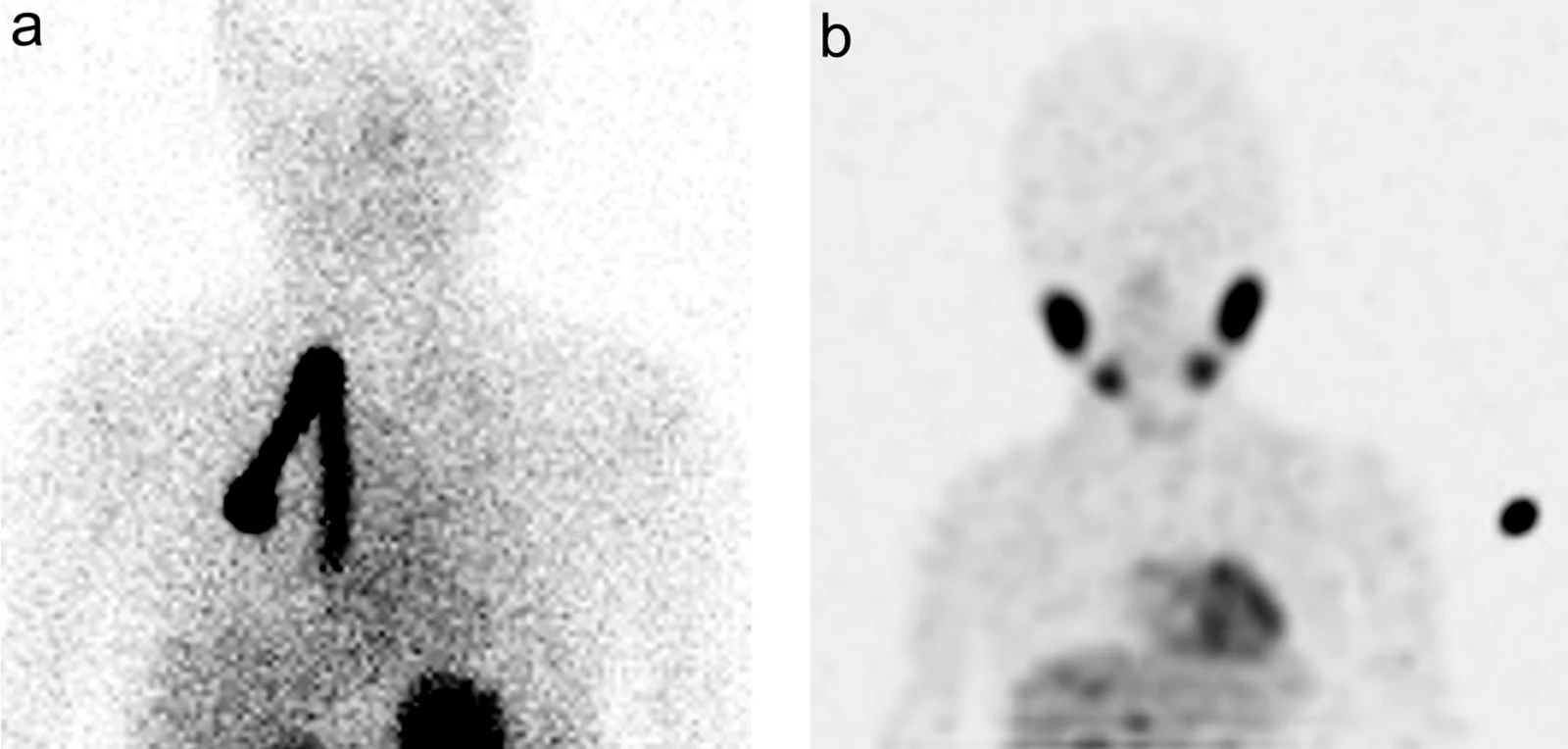
Patient images showing **a**) 4 MBq (6.5% of administered activity) [^99m^Tc]Tc-DMSA residual in an implantable venous port that was outside the clinical field of view and deemed to not interfere with the clinical review and **b**) 1 MBq (0.8% of administered activity) [^123^I]MIBG residual in parts of a peripherally inserted central catheter-line system outside the patient but nothing visible within the patient

**Fig. 6:**
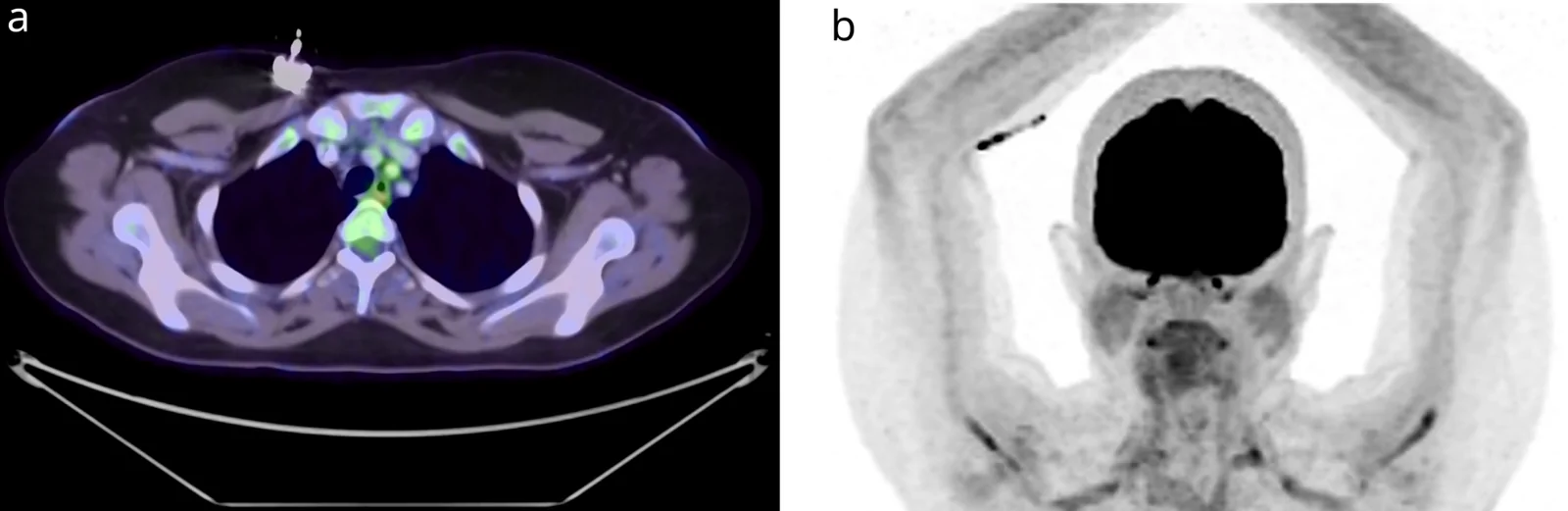
Patient images showing [^18^F]FDG residual in **a**) an implantable venous port and **b**) a peripheral intravenous catheter. In both cases, the residual activity was measured to be < 0.05 MBq

Only 3.3% (7/214) of measurements demonstrated a residual activity fraction exceeding 5% (Table 2). The highest residual absolute activity was 31 MBq ([^99m^Tc]pertechnetate in a PIVC, 4.5% residual activity fraction), and the highest proportional residual activity fraction was 17.3% (19 MBq [^99m^Tc]Tc-MAA in a midline) (Table 2; Fig. 4b). Illustrative patient images showing retained activity across different venous access devices and radiopharmaceuticals are presented in Figs. 4, 5 and 6.

**Table 2:** Patient administrations with more than 5% residual activity fraction or 5 MBq residual activity

Radiopharmaceutical	Venous access device	NaCl 0.9% flush volume (ml)	Injected activity (MBq)	Residual activity (MBq)	Residual activity fraction (%)
[^99m^Tc]Tc-MAA	Midline	50	112	19	17.3%
[^99m^Tc]Tc-MAA	PIVC	20	82	8	9.5%
[^99m^Tc]Tc-MAA	Nexiva	35	125	9	7.2%
[^99m^Tc]Tc-DMSA	Implanted venous port	30	59	4	6.5%
[^99m^Tc]Tc-MBrIDA	PIVC	15	21	1	6.4%
[^99m^Tc]pertechnetate	PIVC	10	40	2	5.5%
[^99m^Tc]Tc-MAA	PIVC	20	117	6	5.4%
[^99m^Tc]Tc-MAA	Deep Access	50	122	6	4.9%
[^99m^Tc]pertechnetate	PIVC	10	692	31	4.5%
[^99m^Tc]Tc-tetrofosmin	PIVC	0	245	10	4.2%
[^99m^Tc]pertechnetate	Stopcock	10	691	19	2.7%
[^99m^Tc]pertechnetate	Stopcock	10	387	8	2.0%
[^99m^Tc]pertechnetate	Stopcock	10	363	6	1.7%

### Clinical implications

Out of the 167 patient administrations, the venous access device was within the clinical field of view in 84 cases (50%). Although residual activity was visually detectable either within the patient (in the tubing) or outside the patient (in the injection system; an example is provided in Fig. 5b) in some instances, 30 of 84 cases (36%), no cases were identified in which residual activity interfered with or altered the clinical image interpretation, either at original clinical review or during the study-specific image assessment. Patients in whom the PIVCs had been removed prior to imaging were not included in the clinical assessment.

### Experimental setting

In the experimental setup, 45 venous access devices and an equal number of stopcocks were tested, yielding a total of 90 measurements, with eight different radiopharmaceuticals. The datasets for vial measurements are outlined in Supplementary Material 3b. For the vial administrations, only 3.3% (3/90) of measurements exceeded a 5% residual activity fraction limit. All three measurements were of [^99m^Tc]Tc-tetrofosmin retained in stopcocks with highest residual activity fraction and absolute activity of 11.1% and 22 MBq, respectively.

## Discussion

In this consecutive observational real‑world cohort of patients undergoing clinical SPECT and PET imaging at a two‑site university hospital, we systematically evaluated residual activity after radiopharmaceutical injection across commonly used tracers and intravenous access devices. Residual activity was consistently low; only 3.3% of patient injections exceeded the 5% threshold, and the highest residual activity measured was 31 MBq. Importantly, no case showed any impact on image interpretation or subsequent clinical assessment.

These findings carry two main implications. First, they provide practical reassurance that small amounts of residual activity, when present, do not compromise lesion visibility or routine qualitative reporting for either SPECT or PET. Second, the uniformly low residual activity across different venous access routes indicates that flexible intravenous access strategies are feasible in routine clinical practice—including adult and pediatric patients with challenging venous access—without risking diagnostic reliability. Within the present material, only one measurement exceeded the 10% threshold of residual activity, namely one [^99m^Tc]Tc-MAA administration through a midline catheter, i.e. a type of PIVC. Even in this case, no clinically relevant effect on image quality or interpretation was observed. Although only observed in a single case, until confirmed in larger studies, some caution may be warranted regarding [^99m^Tc]Tc-MAA administration across different venous access devices.

Taken together, these results suggest that injection site residual activity seldom interferes with image interpretation and, within the investigated setting, does not support routine administration of additional activity to compensate for residual radiopharmaceuticals.

With the modern widespread availability of SPECT/CT at most nuclear medicine clinics, the potential clinical impact of unexpected focal uptake caused by residual radiopharmaceutical activity has been substantially reduced. The improved anatomical localization provided by SPECT/CT enables such findings to be readily identified, contextualized, and dismissed when appropriate, making residual activity-related artefacts a minor issue in contemporary practice.

### Available guidance on use of venous access devices

PIVCs including midline catheters are generally not recommended for administration of strongly irritant infusions, such as certain cytotoxic drugs, or solutions with markedly high or low pH values. PICCs should not be used for drugs with pH < 5 or > 9, osmolarity > 600 mOsm/L, or agents causing pain upon injection (Region Skåne, Vårdhandboken 2026). CVCs are indicated for patients who are difficult to cannulate and when infusion of irritant or vasoconstrictive agents is needed. Likewise, implantable venous ports are suitable for repeated administration of irritant drugs (e.g., chemotherapy), total parenteral nutrition, and long-term intravenous therapy or sampling in patients with poor venous access (Region Skåne, Vårdhandboken 2026). However, to the best of our knowledge, there are no specific guidelines addressing the administration of radiopharmaceuticals. Moreover, during the systematic literature review, no clinical studies for the evaluation of uptake in PIVCs, PICCc, CVCs, or implanted venous ports could be identified. For commercially available radiopharmaceuticals, the summaries of product characteristics jointly report the lowest pH range of 2.3–3.5 and the highest range of 7.5–9.0. Data on osmolality is generally absent, and administration instructions are limited and inconsistently reported. Only a single product (Pulmocis) explicitly advises against administration via implanted venous ports (CIS bio international 2019) (Supplementary Material 4).

### Comparison to previous studies

Previous studies reported minimal residual activity in PIVCs after bone scintigraphy, with an average residual activity of 0.04% in 29 patients (Miozza et al. 2024) and 0.12 MBq in 48 patients (Warminski et al. 2019). In contrast, in the current study, the maximal residual activity fraction and highest retained activity for [^99m^Tc]Tc-DPD and [^99m^Tc]Tc-HDP across the seventeen PIVCs were 0.6% (0.1 MBq) and 1.0 MBq (0.2%), respectively. However, all values remained well below levels likely to result in clinically relevant consequences.

Proper injection technique and flushing with NaCl 0.9% impacts residual activity fraction in various settings within nuclear medicine practices (Gossman et al. 2017, Drescher et al. 2021). In the laboratory study by Gossman et al., the residual activity fraction of [^18^F]FDG and Na[^18^F]F in various implantable venous ports using in micro-PET imaging was found to be < 1% following a 10 ml NaCl 0.9% flush (Gossman et al. 2017). Here, injections with [^18^F]FDG followed by a NaCl 0.9% flush of 10 ml or more resulted in less than 0.5% residual activity.

In the case reports by Slavin et al. and Becherer et al. (Becherer et al. 1998, Slavin et al. 2004), respectively, the residual activity of administered radiopharmaceuticals ([^99m^Tc]Tc-MIBI and an unspecified bone scintigraphy tracer, respectively) in central venous catheters initially caused a clinical conundrum. This issue was resolved through repeated imaging and continuous flushing with NaCl 0.9%. In both cases, SPECT/CT was not utilized (reports published in the year 1998 and 2004, respectively), and would likely have resolved the issue.

### Local tissue effects

Extravasation, the inadvertent administration of a radiopharmaceutical into the tissue surrounding a peripheral vein, is a well-known complication in clinical practice (Harris et al. 2023). Although it typically results in minor adverse effects, in severe cases it may lead to significant tissue damage due to the localized delivery of a high radiation dose. Although the present study investigates residual activity within venous access devices rather than extravasation, both phenomena involve unintended residual radiopharmaceutical activity and are therefore relevant from a local radiation safety perspective. In a large retrospective multicenter study evaluating injection site dose infiltration across ten imaging centers and 1,000 patients undergoing PET across five different tracers, no patient exhibited more than 1% of the injected activity remaining at the injection site (Sunderland et al. 2023). In our cohort, given the ^18^F-, ^68^Ga-, ^99m^Tc-, and ^123^I-labeled radiopharmaceuticals and the low levels of residual activity within the venous access devices, local tissue injury is not expected to occur (Van der Pol et al. 2017). Due to the higher administered dose and isotope properties, extravasation poses a larger problem in therapeutic nuclear medicine than it does in diagnostic nuclear medicine (Arveschoug et al. 2020).

### Person-centered care

Our descriptive findings support the use of existing venous access devices for radiopharmaceutical administration, potentially reducing the need for new PIVC placement and associated complications, including pain, hematoma, thrombophlebitis, and infection. Incorporating patient preferences and providing advance information on venous access may further reduce procedure-related distress, particularly in pediatric populations (Runge et al. 2025). In addition, the use of existing venous access may facilitate workflow for both referring wards and the nuclear medicine department, improving procedural efficiency and overall patient experience.

### When not to use pre‑existing vascular access

There are circumstances in which inserting a new PIVC is strongly recommended. In hospitalized patients undergoing [^18^F]FDG PET for suspected or confirmed infection, reusing a previously placed venous access device may complicate image interpretation. Local inflammation or infection associated with an indwelling or recently used PIVC, including thrombophlebitis, can lead to focal [^18^F]FDG uptake at or near the catheter site, thereby mimicking radiotracer residual activity. To enhance diagnostic confidence, a new PIVC should be placed before imaging, accompanied by clear documentation indicating whether the venous access device is newly inserted or pre-existing.

### New knowledge gained

Our findings provide a first descriptive overview of residual radiopharmaceutical activity in different venous access devices during routine SPECT and PET imaging. While the study was not powered to generate robust statistical estimates, the observed patterns consistently demonstrated a very low level of residual activity. Nevertheless, the results also indicate that variability exists across types of venous access devices and tracers, underscoring the need for further studies to clarify the technical and clinical relevance of injection-site residual activity.

### Limitations

The study was conducted at a single institution, bi-center hospital, which may limit external validity. In accordance with the ethical approval, no patient-level clinical characteristics were recorded. Consequently, variables such as patient age, venous access history, and comorbidities could not be analyzed. Data on age of venous access device, number of lumens, prior complications, and catheter related adverse events were not systematically recorded, and catheter related complications were not monitored as part of the study protocol. Furthermore, potential technical explanations for isolated cases of elevated retained activity, such as catheter malposition, partial obstruction or clotting, or injection resistance were not evaluated. This is particularly relevant for the single [^99m^Tc]Tc-MAA administration through a midline catheter that demonstrated higher residual activity than the remaining cases.

Anatomical venous access sites may influence residual radiopharmaceutical activity. Distal injections (e.g., hand or wrist) may increase transit time and partial stasis compared with proximal sites (e.g., antecubital vein). However, site-specific analysis was not feasible. The potential influence of CT contrast agents was not evaluated, and any interaction between iodinated contrast administration and radiopharmaceutical distribution or local venous residual activity could therefore not be assessed. Complications related to venous access device (e.g., clogging or membrane damage) were not assessed in this study.

The use of static imaging limits sensitivity for detecting very low radiotracer residual activity fraction, particularly in the presence of high background activity, potentially introducing uncertainty in estimating catheter-related effects. Residual activity may also be underestimated in static images due to attenuation within the patient. However, as most components of the injection system are superficially located and partly external, attenuation effects are likely limited, allowing a reasonable estimation of residual activity. Furthermore, uncertainties in the quantification of low activity levels may have affected the precision of the results. A potential effect of residual PET tracer activity on standardized uptake value (SUV) quantification was not formally evaluated; however, given the very low residual activity fractions observed, any impact on SUV measurements is expected to be negligible. Removable PIVCs and venous access devices in the experimental setup were measured in an activity meter with isotope factors calibrated for a wide range of vial and syringe geometries. Although these devices are not a calibrated geometry, the limited variation of only a few percent between calibrated geometries for the used isotopes suggests that the clinical calibration factors are applicable for the measurements.

In addition to the review of routine clinical reports, the study-specific image review was performed by a single non-blinded reader.

Conclusions are limited to the studied radiopharmaceuticals, with no generalization beyond these agents. Therapeutic radiopharmaceuticals were outside the scope of this study, as their administration is handled by a separate clinical department at our hospital. Furthermore, the impact of venous access device on systemic pharmacokinetics, such as alterations in blood activity curves or tracer recirculation, was not investigated.

## Conclusion

In this descriptive real-world cohort study, we conducted an exploratory assessment of residual radiopharmaceutical activity in different venous access devices during SPECT and PET imaging. Overall, residual activity fractions were low across the radiopharmaceuticals and venous access systems evaluated, with only a minority of cases exceeding 5% of the administered activity. Importantly, the observed residual activity did not interfere with visual image interpretation in the reviewed examinations. Prospective studies across a broader range of radiopharmaceuticals and venous access devices, with inclusion of quantitative endpoints, are required to more comprehensively define the clinical implications of residual radiopharmaceutical activity in venous access devices.

## Supplementary Information

Below is the link to the electronic supplementary material.


Supplementary Material 1. An illustrative overview of the included venous access devices and representative vascular access routes



Supplementary Material 2. Literature search report



Supplementary Material 3. Tables of measurement data from (a) patient administrations and (b) vial administrations. Residual activities < 0.5 MBq and residual activity fractions < 0.05% have been rounded to zero



Supplementary Material 4. Information provided in the summaries of product characteristics for clinically used radiopharmaceuticals at study site


## Data Availability

The data are not publicly available due to restrictions by Swedish and European law to protect patient privacy. The raw datasets for the experimental setup are available from the corresponding author on reasonable request.

## Abbreviations

ALARA: As low as reasonably achievable
CT: Computed tomography
CVC: Central venous catheter
PACS: Picture archiving and communication system
PET: Positron emission tomography
PICC: Peripherally inserted central catheter
PIVC: Peripheral intravenous catheter
ROI: Region-of-Interest
SPECT: Single-photon emission computed tomography
SUV: Standardized uptake value
VOI: Volume-of-Interest
