# Supplementary material for: Residual radiopharmaceutical activity in venous access devices during PET and SPECT imaging: a real-world clinical observational cohort study
Source: EJNMMI Rep. 2026 Sep 1;10(1):33. doi: 10.1186/s41824-026-00317-4 (PMC13529631; doi:10.1186/s41824-026-00317-4)
Supplement: Supplementary file 1 [file 41824_2026_317_MOESM2_ESM.pdf]

LUND UNIVERSITY
Faculty of Medicine

# SUPPLEMENTARY MATERIAL 2.

Literature search report

## RESEARCH QUESTION, AIM WITH LITERATURE SEARCH

1. How much radiopharmaceutical gets trapped in venous catheters during SPECT and PET imaging?
2. How do different radiopharmaceuticals differ regarding the question in point 1?
3. What is the clinical significance of radiopharmaceuticals getting trapped?

### PubMed (National Library of Medicine)

**Date of search: March 11, 2025**

#1
((("Radiopharmaceuticals"[Mesh]) OR "Tomography, Emission-Computed, Single-Photon"[Mesh]) OR "Radionuclide Imaging"[Mesh]) OR "Positron Emission Tomography Computed Tomography"[Mesh]
=256140

#2
Tc-mag3[Title/Abstract] OR TC-99m MAG3[Title/Abstract] OR technescan[Title/Abstract] OR TC-Maa[Title/Abstract] OR Tc-Hdp[Title/Abstract] OR technetium[Title/Abstract] OR TC-Brida[Title/Abstract] OR Tc-pertechnatate[Title/Abstract] OR I-mIBG[Title/Abstract] OR Tc-mioview[Title/Abstract] OR F-FDG[Title/Abstract] OR F-PSMA[Title/Abstract] OR F-mFBG[Title/Abstract] OR F-Choline[Title/Abstract] OR F-DOPA[Title/Abstract] OR F-flutemetamol[Title/Abstract] OR F-Tau[Title/Abstract] OR Ga-DOTATOC[Title/Abstract] OR Ga-PMSA[Title/Abstract] OR radiopharmaceutical*[Title/Abstract] OR SPECT[Title/Abstract] OR Single-Photon emission-computed tomography[Title/Abstract] OR scintigraphy[Title/Abstract] OR radionuclide imaging[Title/Abstract] OR PET[Title/Abstract] OR positron emission tomography[Title/Abstract]
=255921

#3
#1 OR #2
=359020

#4
"Central Venous Catheters"[Mesh] OR "Vascular Access Devices"[Mesh] OR “Catheters”[Mesh]
=39472

#5
PIVC[Title/Abstract] OR peripheral intervenous catheter[Title/Abstract] OR vascular access port*[Title/Abstract] OR peripheral venous catheter[Title/Abstract] OR PVK[Title/Abstract] OR Deep access[Title/Abstract] OR midline[Title/Abstract] OR central venous catheter[Title/Abstract] OR CVK[Title/Abstract] OR peripherally inserted central catheter[Title/Abstract] OR PICC[Title/Abstract] OR nexiva[Title/Abstract] OR closed 1V catheter system[Title/Abstract] OR venous port*[Title/Abstract] OR portacath[Title/Abstract] OR port catheter*[Title/Abstract]
=55787

#6
#4 OR #5
=90546

#7
"Radioactivity"[Mesh]
=7187

#8
radioactivity OR radioactive
=134110

#9
#7 OR #8
=134110

#10
retent* OR remain* OR linger* OR residual OR measurement
=7407195

#11
#9 AND #10
=42345

#12
#3 AND #6 AND #11
=**21 records**

## Embase.com (Elsevier, 1947-present)

**Date of search: March 11, 2025**

#1
'radiopharmaceutical agent'/exp OR 'single photon emission computed tomography'/exp OR 'scintiscanning'/exp OR 'positron emission tomography'/exp OR 'tc mag3':ti,ab,kw OR 'tc-99m mag3':ti,ab,kw OR technescan:ti,ab,kw OR 'tc maa':ti,ab,kw OR 'tc hdp':ti,ab,kw OR technetium:ti,ab,kw OR 'tc brida':ti,ab,kw OR 'tc pertechnatate':ti,ab,kw OR 'i mibg':ti,ab,kw OR 'tc mioview':ti,ab,kw OR 'f fdg':ti,ab,kw OR 'f psma':ti,ab,kw OR 'f mfbg':ti,ab,kw OR 'f choline':ti,ab,kw OR 'f dopa':ti,ab,kw OR 'f flutemetamol':ti,ab,kw OR 'f tau':ti,ab,kw OR 'ga dotatoc':ti,ab,kw OR 'ga pmsa':ti,ab,kw OR radiopharmaceutical*:ti,ab,kw OR spect:ti,ab,kw OR 'single-photon emission-computed tomography':ti,ab,kw OR scintigraphy:ti,ab,kw OR 'radionuclide imaging':ti,ab,kw OR pet:ti,ab,kw OR 'positron emission tomography':ti,ab,kw

=750015

#2
'vascular access device'/exp OR 'central venous catheter'/exp OR 'catheters'/exp OR pivc:ti,ab,kw OR 'peripheral intervenous catheter':ti,ab,kw OR 'vascular access port*':ti,ab,kw OR 'peripheral venous catheter':ti,ab,kw OR pvk:ti,ab,kw OR 'deep access':ti,ab,kw OR midline:ti,ab,kw OR 'central venous catheter':ti,ab,kw OR cvk:ti,ab,kw OR 'peripherally inserted central catheter':ti,ab,kw OR picc:ti,ab,kw OR nexiva:ti,ab,kw OR 'closed 1v catheter system':ti,ab,kw OR 'venous port*':ti,ab,kw OR portacath:ti,ab,kw OR 'port catheter*':ti,ab,kw
=356565

#3
'radioactivity'/exp OR radioactivity OR radioactive
=19527

#4
retent* OR remain* OR linger* OR residual OR 'measurement'/exp OR measurement
=7009900

#5
#3 AND #4
=51440

#6
#1 AND #2 AND #4
=**219 records**

## INSPEC

**Date of search: March 11, 2025**

#1
(((((Tc-mag3 OR TC-99m MAG3 OR technescan OR TC-Maa OR Tc-Hdp OR technetium OR TC-birds OR Tc-pertechnetate OR I-mIBG OR Tc-movie OR F-FDG OR F-PSMA OR F-mfg OR F-Choline OR F-DOPA OR F-flutemetamol OR F-Tau OR Ga-domatic OR Ga-pmma OR radiopharmaceutical* OR SPECT OR Single-Photon emission-computed tomography OR scintigraphy OR radionuclide imaging OR PET OR positron emission tomography) WN ALL) AND ((pvc OR peripheral intravenous catheter OR vascular access port* OR peripheral venous catheter OR PVK OR Deep access OR midline OR central venous catheter OR CVK OR peripherally inserted central catheter OR PICC OR nerva OR closed 1V catheter system OR venous port* OR portacath OR port catheter*) WN ALL)) AND ((radioactivity OR radioactive) WN ALL)) AND ((retention OR residual OR remain OR linger) WN ALL))
=**0 records**

---

## Total number of references from database searches: 240 records

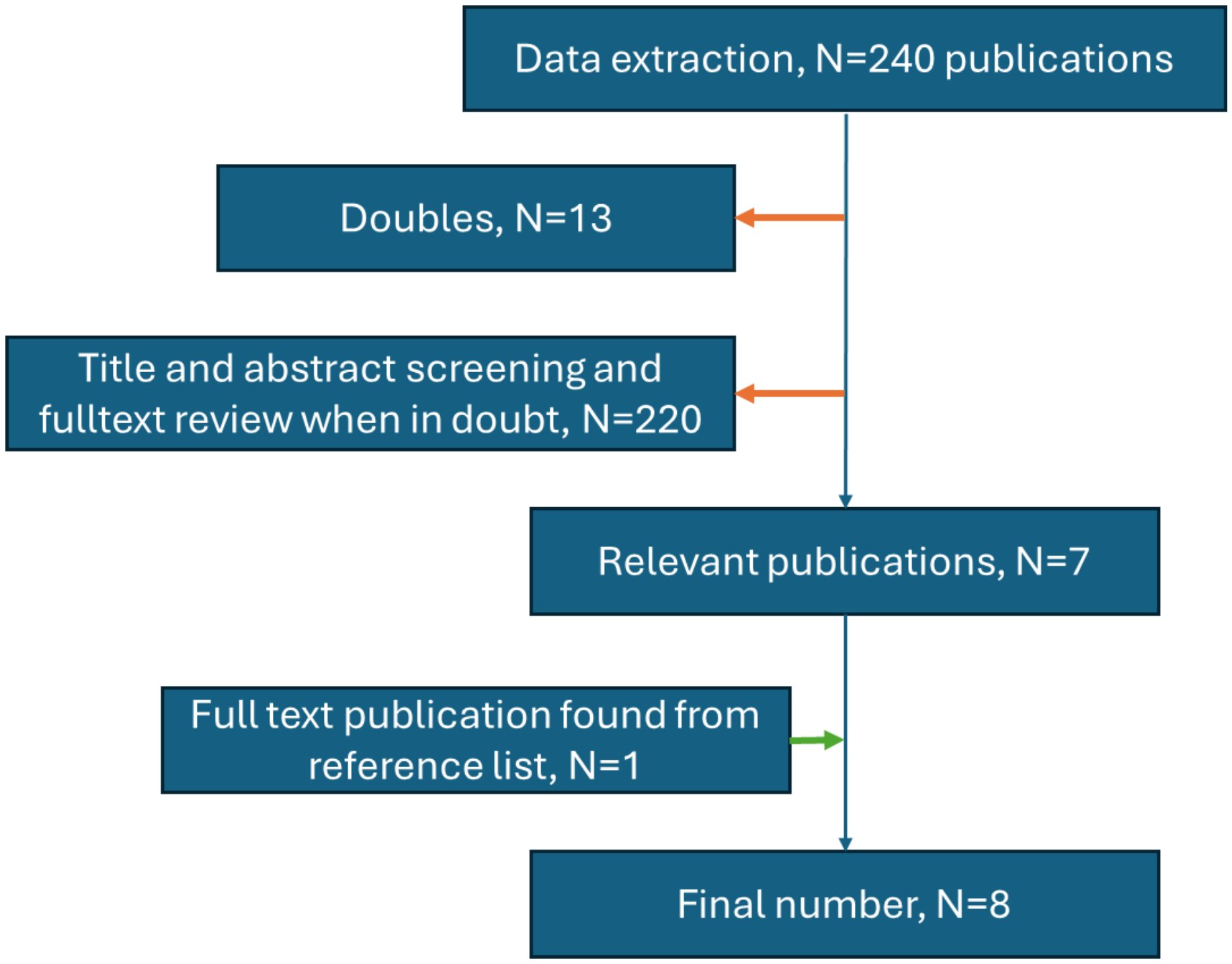


*Figure 1 Flow chart of identified publications*

*Table 1. List of the eight articles identified through the systematic literature search*

| | | |
|---|---|---|
| 1 | Case report/interesting images | Becherer, A., Mentes, M., & Leitha, T. (1998). Tracer retention in a central venous catheter [Article]. *Clinical Nuclear Medicine*, *23*(1), 45-46. https://doi.org/10.1097/00003072-199801000-00017 |
| 2 | For methodological comparisons. | Drescher, R., Seifert, P., Gühne, F., Aschenbach, R., Kühnel, C., & Freesmeyer, M. (2021). Radioembolization With Holmium-166 Polylactic Acid Microspheres: Distribution of Residual Activity in the Delivery Set and Outflow Dynamics During Planning and Treatment Procedures [Article]. *Journal of Endovascular Therapy*, *28*(3), 452-462. https://doi.org/10.1177/1526602821996719 |
| 3 | Considered most relevant article for comparison | Gossman, M. S., Zheng, H., Evans, J. G., Li, J., & Ng, C. K. (2017). Assessment of radiopharmaceutical retention for vascular access ports using positron emission tomography imaging. *J Appl Clin Med Phys*, *18*(6), 244-249. https://doi.org/10.1002/acm2.12196 |
| 4 | Sparsely described, conference abstract | Jacob, A. (2022). Introducing tracer injections through Portacaths Indre Vaitkute, Katharine Chalmers, Sorcha Curry [Conference Abstract]. *Nuclear Medicine Communications*, *43*(5), 591. https://doi.org/10.1097/MNM.0000000000001555 |
| 5 | Case report/interesting images | Slavin Jr, J. D., Vetter, C. L., & Spencer, R. P. (2004). "Disappearing" mediastinal parathyroid adenoma: Retention at the tip of an intrathoracic catheter [Article]. *Clinical Nuclear Medicine*, *29*(11), 734. https://doi.org/10.1097/00003072-200411000-00018 |
| 6 | Clinically meaningful infiltration events, absorbed dose to skin | Sunderland, J., York, D., Graves, S., Mundt, C., & Bartel, T. (2024). Frequency and significance of injection infiltration and associated dosimetry in clinical PET/CT: A multi-center investigation [Conference Abstract]. *Clinical Nuclear Medicine*, *49*(5), e238-e239. https://www.embase.com/search/results?subaction=viewrecord&id=L644259480&from=export<br>the full text article: https://pmc.ncbi.nlm.nih.gov/articles/PMC10586484/<br>Multicenter Evaluation of Frequency and Impact of Activity Infiltration in PET Imaging, Including Microscale Modeling of Skin-Absorbed Dose \| Journal of Nuclear Medicine |
| 7 | Measurement in vitro of IV catheters | Warminski, J., Gruchot, M., Herrera, M., Gersman, J., & Dillehay, G. (2019). Evaluation of Tc-99m MDP residual activity in IV catheters [Conference Abstract]. *Journal of Nuclear Medicine*, *60.* https://www.embase.com/search/results?subaction=viewrecord&id=L629439226&from=export |
| 8 | Abstract identified from article nr 7. | Examining residual radioactivity of IV catheters utilized in nuclear medicine whole-body bone imaging. \| Journal of Nuclear Medicine |
