# Supplementary material for: Residual radiopharmaceutical activity in venous access devices during PET and SPECT imaging: a real-world clinical observational cohort study
Source: EJNMMI Rep. 2026 Sep 1;10(1):33. doi: 10.1186/s41824-026-00317-4 (PMC13529631; doi:10.1186/s41824-026-00317-4)
Supplement: Supplementary file 2 [file 41824_2026_317_MOESM1_ESM.docx]

Supplementary Material 1. An illustrative overview of the included venous access devices and representative vascular access routes.

The figure illustrates representative insertion sites and approximate vascular pathways. The image was generated using ChatGPT as a schematic illustrative aid to improve clarity and understanding of the different devices and vascular access routes. The figure is not drawn to scale and does not claim exact anatomical correctness.
