# Supplementary material for: Residual radiopharmaceutical activity in venous access devices during PET and SPECT imaging: a real-world clinical observational cohort study
Source: EJNMMI Rep. 2026 Sep 1;10(1):33. doi: 10.1186/s41824-026-00317-4 (PMC13529631; doi:10.1186/s41824-026-00317-4)
Supplement: Supplementary file 3 [file 41824_2026_317_MOESM3_ESM.docx]

Supplementary Material 3a) Measurement data from patient administrations. Residual activities <0.5 MBq and residual activity fractions <0.05% have been rounded to zero.

| Radiopharmaceutical | Venous access devices or injection component | Number of administrations | Mean NaCl 0.9% flush volume (Min-Max) (ml) | Mean injected activity (Min-Max) (MBq) | Mean residual activity (Min-Max) (MBq) | Mean residual activity fraction (Min-Max) (%) |
| --- | --- | --- | --- | --- | --- | --- |
| [^18^F]FDG |  | 30 | 73 (10 - 245) | 184 (21 - 421) | 0 (0 - <0.5) | 0 (0 – 0.3) |
|  | PIVC | 7 | 102 (35 - 199) | 252 (165 - 421) | 0 (0 - <0.5) | 0 (0 - <0.05) |
|  | Midline | 1 | 35 (35 - 35) | 233 (233 - 233) | 0 (0 - <0.5) | 0 (0 - <0.05) |
|  | PICC-line | 12 | 67 (10 - 224) | 206 (57 - 379) | 0 (0 - <0.5) | 0 (0 - <0.05) |
|  | Implanted venous port | 10 | 64 (10 - 245) | 105 (21 - 230) | 0 (0 - <0.5) | 0.1 (0 – 0.3) |
| [^18^F]fluorocholine |  | 40 | 16 (10 - 20) | 256 (180 - 360) | 0 (0 - <0.5) | 0 (0 - 0.2) |
|  | PIVC | 20 | 16 (10 - 20) | 256 (180 - 360) | 0 (0 - <0.5) | 0 (0 - 0.1) |
|  | Stopcock | 20 | 16 (10 - 20) | 256 (180 - 360) | 0 (0 - <0.5) | 0 (0 - 0.2) |
| [^68^Ga]Ga-DOTA-TOC |  | 11 | 191 (101 - 214) | 126 (88 - 169) | 0 (0 - <0.5) | 0 (0 - 0.1) |
|  | PIVC | 6 | 184 (101 - 214) | 123 (88 - 169) | 0 (0 - <0.5) | 0 (0 - 0.1) |
|  | Stopcock | 5 | 200 (190 - 214) | 130 (105 - 169) | 0 (0 - <0.5) | 0 (0 - 0.1) |
| [^68^Ga]Ga-PSMA-11 |  | 4 | 10 (10 - 10) | 125 (112 - 138) | 0 (0 - <0.5) | 0 (0 - 0.1) |
|  | PIVC | 2 | 10 (10 - 10) | 125 (112 - 138) | 0 (0 - <0.5) | 0 (0 - 0.1) |
|  | Stopcock | 2 | 10 (10 - 10) | 125 (112 - 138) | 0 (0 - <0.5) | 0 (0 - <0.05) |
| [^99m^Tc]Tc-DMSA |  | 13 | 12 (5 - 40) | 59 (19 - 126) | 0 (0 - 4) | 0.9 (0.1 - 6) |
|  | PIVC | 8 | 7 (5 - 10) | 51 (19 - 126) | 0 (0 - <0.5) | 0.4 (0.2 - 1) |
|  | CVC | 1 | 40 (40 - 40) | 22 (22 - 22) | 0 (0 - <0.5) | 1 (1 - 1) |
|  | Stopcock | 3 | 10 (10 - 10) | 93 (73 - 126) | 0 (0 - <0.5) | 0.3 (0.1 - 0.5) |
|  | Implanted venous port | 1 | 30 (30 - 30) | 59 (59 - 59) | 4 (4 - 4) | 6 (6 - 6) |
| [^99m^Tc]Tc-DPD |  | 9 | 17 (7 - 40) | 562 (490 - 615) | 1 (0 - 2) | 0.1 (0 - 0.4) |
|  | PIVC | 6 | 9 (7 - 10) | 587 (558 - 615) | 1 (0 - 1) | 0.1 (0 - 0.2) |
|  | Nexiva | 1 | 40 (40 - 40) | 554 (554 - 554) | 2 (2 - 2) | 0.4 (0.4 - 0.4) |
|  | Stopcock | 1 | 30 (30 - 30) | 490 (490 - 490) | 0 (0 - <0.5) | 0 (0 - <0.05) |
|  | Implanted venous port | 1 | 30 (30 - 30) | 490 (490 - 490) | 0 (0 - <0.5) | 0 (0 - <0.05) |
| [^99m^Tc]Tc-HDP |  | 17 | 12 (10 - 30) | 482 (14 - 609) | 0 (0 - 1) | 0.1 (0 - 0.6) |
|  | PIVC | 11 | 12 (10 - 30) | 480 (14 - 585) | 0 (0 - 1) | 0.1 (0 - 0.6) |
|  | Bionector | 2 | 20 (10 - 30) | 288 (14 - 562) | 0 (0 - <0.5) | 0.2 (0 - 0.4) |
|  | Deep Access | 1 | 10 (10 - 10) | 609 (609 - 609) | 0 (0 - <0.5) | 0 (0 - <0.05) |
|  | Stopcock | 3 | 10 (10 - 10) | 577 (566 - 585) | 0 (0 - <0.5) | 0 (0 - <0.05) |
| [^99m^Tc]Tc-MAA |  | 26 | 27 (10 - 50) | 114 (18 - 151) | 2 (0 - 19) | 2 (0 - 17) |
|  | PIVC | 17 | 20 (10 - 28) | 112 (18 - 151) | 1 (0 - 8) | 1 (0 - 10) |
|  | CVC | 2 | 35 (30 - 40) | 120 (116 - 123) | 1 (1 - 1) | 0.7 (0.5 - 0.9) |
|  | Deep Access | 2 | 43 (35 - 50) | 122 (122 - 122) | 3 (0 - 6) | 3 (0.2 - 5) |
|  | Midline | 1 | 50 (50 - 50) | 112 (112 - 112) | 19 (19 - 19) | 17 (17 - 17) |
|  | Nexiva | 3 | 38 (35 - 40) | 114 (107 - 125) | 3 (0 - 9) | 3 (0.1 - 7) |
|  | PICC-line | 1 | 40 (40 - 40) | 123 (123 - 123) | 1 (1 - 1) | 0.5 (0.5 - 0.5) |
| [^99m^Tc]Tc-MAG3 |  | 12 | 14 (10 - 19) | 51 (23 - 79) | 0 (0 - <0.5) | 0.2 (0 - 2) |
|  | PIVC | 10 | 15 (10 - 19) | 56 (23 - 79) | 0 (0 - <0.5) | 0.1 (0 - 0.2) |
|  | Bionector | 1 | 11 (11 - 11) | 26 (26 - 26) | 0 (0 - <0.5) | 0 (0 - <0.05) |
|  | Stopcock | 1 | 11 (11 - 11) | 26 (26 - 26) | 0 (0 - <0.5) | 2 (2 - 2) |
| [^99m^Tc]Tc-MBrIDA |  | 5 | 11 (4 - 15) | 18 (16 - 21) | 0 (0 - 1) | 2 (0.1 - 6) |
|  | PIVC | 1 | 15 (15 - 15) | 21 (21 - 21) | 1 (1 - 1) | 6 (6 - 6) |
|  | CVC | 3 | 10 (4 - 15) | 18 (16 - 19) | 0 (0 - <0.5) | 0.7 (0.1 - 2) |
|  | Implanted venous port | 1 | 10 (10 - 10) | 18 (18 - 18) | 0 (0 - <0.5) | 1 (1 - 1) |
| [^99m^Tc]pertechnetate |  | 14 | 11 (10 - 30) | 373 (40 - 692) | 6 (0 - 31) | 1 (0 - 5) |
|  | PIVC | 8 | 10 (10 - 10) | 353 (40 - 692) | 4 (0 - 31) | 1 (0 - 5) |
|  | Nexiva | 1 | 30 (30 - 30) | 359 (359 - 359) | 3 (3 - 3) | 0.8 (0.8 - 0.8) |
|  | Stopcock | 5 | 10 (10 - 10) | 408 (299 - 691) | 8 (4 - 19) | 2 (1 - 3) |
| [^99m^Tc]Tc-tetrofosmin |  | 6 | 8 (0 - 10) | 292 (226 - 372) | 2 (0 - 10) | 0.7 (0 - 4) |
|  | PIVC | 6 | 8 (0 - 10) | 292 (226 - 372) | 2 (0 - 10) | 0.7 (0 - 4) |
| [^123^I]MIBG |  | 27 | 31 (20 - 60) | 121 (74 - 174) | 0 (0 - 2) | 0.2 (0 - 1) |
|  | PIVC | 6 | 21 (20 - 25) | 115 (106 - 127) | 0 (0 - 1) | 0.2 (0 - 0.4) |
|  | Bionector | 4 | 33 (30 - 40) | 130 (74 - 174) | 0 (0 - <0.5) | 0 (0 - <0.05) |
|  | PICC-line | 6 | 32 (30 - 40) | 111 (75 - 174) | 1 (0 - 2) | 0.5 (0 - 1) |
|  | Stopcock | 1 | 40 (40 - 40) | 169 (169 - 169) | 0 (0 - <0.5) | 0 (0 - <0.05) |
|  | Implanted venous port | 10 | 35 (20 - 60) | 123 (74 - 170) | 0 (0 - 1) | 0.2 (0 - 0.8) |
| All radiopharmaceuticals | All measurements | 214 | 35 (0 - 245) | 213 (14 - 692) | 1 (0 - 31) | 0.5 (0 - 17) |
| All PET-radiopharmaceuticals | All measurements | 85 | 58 (10 - 245) | 208 (21 - 421) | 0 (0 - <0.5) | 0 (0 - 0.3) |
| All SPECT-radiopharmaceuticals | All measurements | 129 | 19 (0 - 60) | 217 (14 - 692) | 1 (0 - 31) | 0.8 (0 - 17) |

Supplementary Material 3b. Measurement data from vial administrations. Residual activities < 0.5 MBq and residual activity fractions < 0.05% have been rounded to zero.

| Radiopharmaceutical | Venous access device or injection component | Number of administrations | Mean NaCl 0.9% flush volume (Min-Max) (ml) | Mean injected activity (Min-Max) (MBq) | Mean residual activity (Min-Max) (MBq) | Mean residual activity fraction (Min-Max) (%) |
| --- | --- | --- | --- | --- | --- | --- |
| [^18^F]FDG |  | 10 | 20 (20 - 20) | 205 (189 - 242) | 0 (0 - <0.5) | 0 (0 - <0.05) |
|  | PIVC | 1 | 20 (20 - 20) | 189 (189 - 189) | 0 (0 - <0.5) | 0 (0 - <0.05) |
|  | Deep Access | 2 | 20 (20 - 20) | 222 (202 - 242) | 0 (0 - <0.5) | 0 (0 - <0.05) |
|  | Midline | 2 | 20 (20 - 20) | 197 (193 - 201) | 0 (0 - <0.5) | 0 (0 - <0.05) |
|  | Stopcock | 5 | 20 (20 - 20) | 205 (189 - 242) | 0 (0 - <0.5) | 0 (0 - <0.05) |
| [^18^F]fluorocholine |  | 4 | 10 (10 - 10) | 227 (218 - 236) | 0 (0.1 - <0.5) | 0.1 (0 - 0.1) |
|  | Deep Access | 2 | 10 (10 - 10) | 227 (218 - 236) | 0 (0.1 - <0.5) | 0.1 (0 - 0.1) |
|  | Stopcock | 2 | 10 (10 - 10) | 227 (218 - 236) | 0 (0.1 - <0.5) | 0.1 (0 - 0.1) |
| [^68^Ga]Ga-PSMA-11 |  | 4 | 10 (10 - 10) | 80 (70 - 90) | 0 (0 - <0.5) | 0.1 (0 - 0.4) |
|  | Deep Access | 1 | 10 (10 - 10) | 70 (70 - 70) | 0 (0 - <0.5) | 0 (0 - <0.05) |
|  | Midline | 1 | 10 (10 - 10) | 90 (90 - 90) | 0 (0 - <0.5) | 0.4 (0.4 - 0.4) |
|  | Stopcock | 2 | 10 (10 - 10) | 80 (70 - 90) | 0 (0 - <0.5) | 0.1 (0 - 0.1) |
| [^99m^Tc]Tc-HDP |  | 12 | 12 (10 - 20) | 533 (399 - 587) | 0 (0 - 2) | 0.1 (0 - 0.4) |
|  | PIVC | 3 | 10 (10 - 10) | 521 (399 - 587) | 0 (0 - <0.5) | 0 (0 - 0.1) |
|  | Deep Access | 2 | 15 (10 - 20) | 538 (497 - 578) | 0 (0 - <0.5) | 0 (0 - <0.05) |
|  | Midline | 1 | 10 (10 - 10) | 560 (560 - 560) | 2 (2 - 2) | 0.4 (0.4 - 0.4) |
|  | Stopcock | 6 | 12 (10 - 20) | 533 (399 - 587) | 0 (0 - 2) | 0.1 (0 - 0.3) |
| [^99m^Tc]Tc-MAA |  | 26 | 21 (10 - 25) | 124 (99 - 144) | 0 (0 - 2) | 0.2 (0 - 1) |
|  | PIVC | 4 | 21 (20 - 25) | 121 (117 - 128) | 0 (0 - 1) | 0.3 (0.1 - 1) |
|  | Deep Access | 4 | 19 (10 - 25) | 132 (118 - 144) | 0 (0 - <0.5) | 0.1 (0 - 0.1) |
|  | Midline | 5 | 22 (20 - 25) | 120 (99 - 130) | 0 (0 - <0.5) | 0.1 (0.1 - 0.1) |
|  | Stopcock | 13 | 21 (10 - 25) | 124 (99 - 144) | 0 (0 - 2) | 0.2 (0 - 1) |
| [^99m^Tc]Tc-MAG3 |  | 8 | 10 (10 - 10) | 73 (70 - 76) | 1 (0 - 3) | 2 (0.1 - 4) |
|  | Deep Access | 2 | 10 (10 - 10) | 72 (71 - 74) | 0 (0 - <0.5) | 0.1 (0.1 - 0.1) |
|  | Midline | 2 | 10 (10 - 10) | 73 (70 - 76) | 0 (0 - <0.5) | 0.1 (0.1 - 0.1) |
|  | Stopcock | 4 | 10 (10 - 10) | 73 (70 - 76) | 2 (2 - 3) | 3 (2 - 4) |
| [^99m^Tc]Tc-tetrofosmin |  | 14 | 10 (10 - 10) | 211 (152 - 265) | 8 (1 - 22) | 4 (0.3 - 11) |
|  | PIVC | 3 | 10 (10 - 10) | 187 (152 - 209) | 3 (2 - 4) | 2 (1 - 2) |
|  | Deep Access | 2 | 10 (10 - 10) | 223 (195 - 251) | 1 (1 - 1) | 0.4 (0.3 - 0.4) |
|  | Midline | 2 | 10 (10 - 10) | 235 (205 - 265) | 8 (8 - 9) | 4 (3 - 4) |
|  | Stopcock | 7 | 10 (10 - 10) | 211 (152 - 265) | 11 (4 - 22) | 5 (2 - 11) |
| [^123^I]MIBG |  | 12 | 18 (10 - 20) | 131 (95 - 243) | 0 (0 - 2) | 0.1 (0 - 1) |
|  | Deep Access | 4 | 18 (10 - 20) | 145 (106 - 243) | 1 (0 - 2) | 0.3 (0 - 1) |
|  | Midline | 2 | 20 (20 - 20) | 103 (95 - 111) | 0 (0 - <0.5) | 0 (0 - 0.1) |
|  | Stopcock | 6 | 18 (10 - 20) | 131 (95 - 243) | 0 (0 - <0.5) | 0 (0 - 0.2) |
| All radiopharmaceuticals | All measurements | 90 | 16 (10 - 25) | 200 (70 - 587) | 1 (0 - 22) | 0.8 (0 - 11) |
| All PET-radiopharmaceuticals | All measurements | 18 | 16 (10 - 20) | 182 (70 - 242) | 0 (0 - <0.5) | 0 (0 - 0.4) |
| All SPECT-radiopharmaceuticals | All measurements | 72 | 16 (10 - 25) | 204 (70 - 587) | 2 (0 - 22) | 1 (0 - 11) |
