# Supplementary material for: Residual radiopharmaceutical activity in venous access devices during PET and SPECT imaging: a real-world clinical observational cohort study
Source: EJNMMI Rep. 2026 Sep 1;10(1):33. doi: 10.1186/s41824-026-00317-4 (PMC13529631; doi:10.1186/s41824-026-00317-4)
Supplement: Supplementary file 4 [file 41824_2026_317_MOESM4_ESM.docx]

Supplementary Material 4. Information provided in the summaries of product characteristics for the used radiopharmaceuticals in this study.

| **Radiopharmaceutical** | **pH** | **Osmolality** | **Instructions for injection** |
| --- | --- | --- | --- |
| [^18^F]FDG | 4.5-8.5 | Not specified |  |
| [^18^F]fluorocholine | 4.5-8.5 | Not specified |  |
| [^68^Ga]Ga-PSMA-11 (Locametz) | 3.2-6.5 | Not specified |  |
| [^68^Ga]Ga-DOTA-TOC (SomaKit TOC) | 3.2-3.8 | Not specified |  |
| [^99m^Tc]Tc-DMSA (Technescan DMSA) | 2.3-3.5 | Not specified |  |
| [^99m^Tc]Tc-DPD (Teceos) | 6.5-7.5 | Not specified |  |
| [^99m^Tc]Tc-HDP (Technescan HDP) | Not specified | Not specified |  |
| [^99m^Tc]Tc-MAA (Pulmocis) | 5.0-7.0 | Not specified | If possible, the product should not be injected via an implanted venous access device, as this can result in inadequate mixing of the radioactivity in the pulmonary artery. |
| [^99m^Tc]Tc-MAG3 (Technescan MAG3) | 5.0-6.0 | Slightly hypertonic |  |
| [^99m^Tc]Tc-MBrIDA (PoltechMBrIDA) | Not specified | Not specified |  |
| [^99m^Tc]pertechnetate | 4-8 | Not specified |  |
| [^99m^Tc]Tc-tetrofosmin (Myoview) | 7.5-9.0 | Not specified |  |
| [^123^I]MIBG | 4.0-5.0 | Not specified | Administered by slow (at least 5 minutes) intravenous injection or by infusion. If desired, the volume to be administered can be increased by dilution. The low pH of the solution may cause injection site pain. Saline flush is recommended following administration. |
